# Suspected Suicidal Cannabis Exposures Reported to US Poison Centers, 2009-2021

**DOI:** 10.1001/jamanetworkopen.2023.9044

**Published:** 2023-04-19

**Authors:** Janessa M. Graves, Julia A. Dilley, Tracy Klein, Erica Liebelt

**Affiliations:** 1 Washington State University College of Nursing, Spokane; 2 Program Design and Evaluation Services, Multnomah County Health Department and Oregon Public Health Division, Portland; 3 Washington State University College of Nursing, Vancouver; 4 Department of Pediatrics, Section of Pediatric Emergency Medicine, Pharmacology & Toxicology, College of Medicine, University of Arkansas for Medical Sciences, Little Rock

## Abstract

This cross-sectional study examines case characteristics of suicidal cannabis exposures reported to US poison centers before vs during the COVID-19 pandemic.

## Introduction

In the US, suicide is a leading cause of death among individuals aged 5 to 64 years.^1^ An association between cannabis use and increased suicidal ideation and attempts has been identified among adolescents and younger adults,^2^ yet not older adults.^3^ Concerns for mental well-being across all ages are increasing, bolstered by increasing suicide rates during the COVID-19 pandemic.^4^ Concurrently, more states have legalized adult-use cannabis.^5^ The purpose of this study was to describe suicidal cannabis exposures reported to US poison centers from January 1, 2009, to December 31, 2021, and compare case characteristics before and during the pandemic.

## Methods

Data were obtained from the National Poison Data System (NPDS) for intentional, suspected suicidal cannabis exposures reported to US poison centers. Poison centers provide free, confidential support to health care professionals and the public for potential exposures and/or adverse reactions to drugs, chemicals, or poisons. This study followed the STROBE reporting guideline, and the Washington State University Office of Research Assurances determined the project to be exempt from the need for institutional review board review because data were deidentified.

Inclusion criteria comprised closed cases of cannabis-related (eTable in Supplement 1) human exposures, age 5 years or older, with reason coded as intentional-suspected suicidal.^6^ Cases missing age or follow-up data were excluded. We compared cases by age group and examined differences by sex, location, and outcomes using χ^2^ tests. We compared 2019 vs 2021 exposures to evaluate potential pandemic effects. Stata/MP, version, 15 software (StataCorp LLC) was used, with α = .05 as the significance threshold; testing was 2-sided.

## Results

From 2009 to 2021, 18 698 intentional, suspected suicidal cannabis exposures (mean [IQR] age, 29 [18-37] years; 51.3% females; 48.7% males) were reported. Exposure counts increased by approximately 17% annually. Most cases occurred in recent years and were among individuals aged 14 to 64 years (Figure). Nearly all (96.5%) cases involved more than 1 substance (Table). Exposures in younger and older age groups occurred more frequently among females. Overall, 9.6% of exposures resulted in death or other major outcomes (ie, life-threatening or with major residual disability or disfigurement).^6^ For older adults, 19.4% of exposures resulted in death or other major outcomes.

**Figure.**
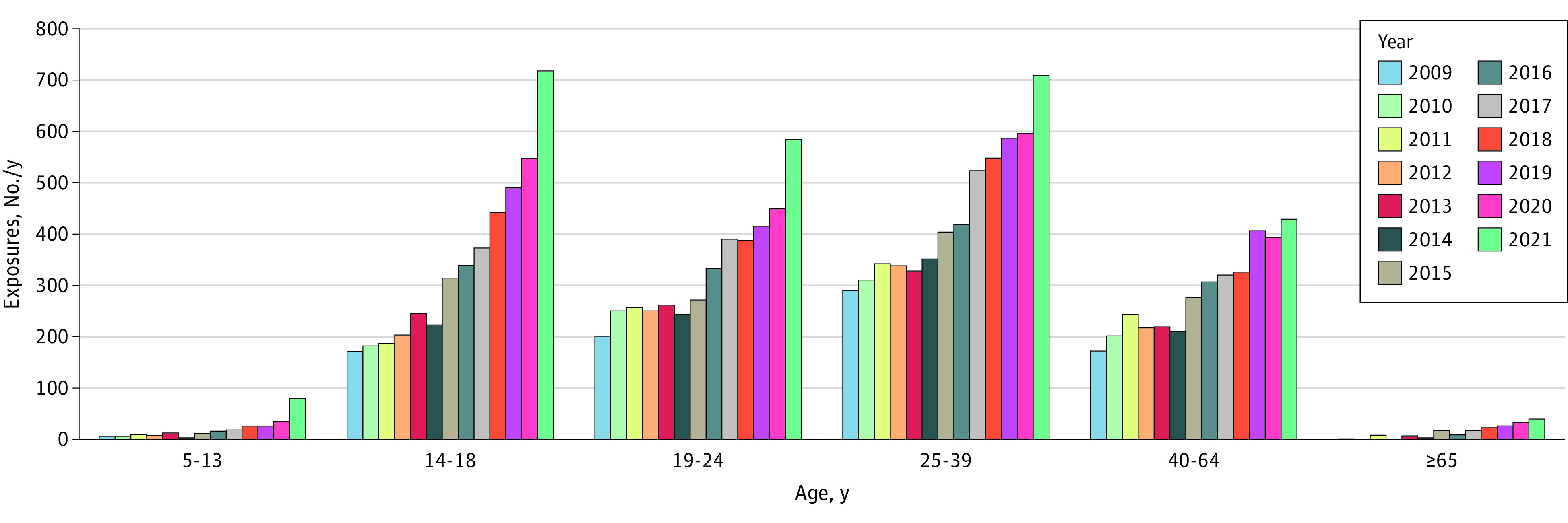
Annual Numbers of Intentional, Suspected Suicidal Cannabis Exposures Reported to US Poison Centers, by Age Group, January 1, 2009, to December 31, 2021

**Table.** Characteristics of Intentional, Suspected Suicidal Cannabis Exposures Reported to US Poison Centers From 2009 to 2021

Characteristic	No. (%) by age group, y^a^						*P* value^b^	All ages, No. (%)
	5-13	14-18	19-24	25-39	40-64	≥65		
No.	256	4448	4307	5758	3733	196	NA	18 698
Sex								
Female	183 (71.5)	2502 (56.3)	2006 (46.6)	2781 (48.3)	2006 (53.7)	110 (56.1)	<.001	9588 (51.3)
Male	73 (28.5)	1944 (43.7)	2299 (53.4)	2977 (51.7)	1727 (46.3)	86 (43.9)		9106 (48.7)
Unknown	0	2 (0)	2 (0)	0	0	0		4 (<0.1)
Medical outcome^c^								
None	30 (11.7)	600 (13.5)	513 (11.9)	623 (10.8)	381 (10.2)	17 (8.7)	<.001	2164 (11.6)
Minor	102 (39.8)	1773 (39.9)	1569 (36.4)	1951 (33.9)	1125 (30.1)	52 (26.5)		6572 (35.1)
Moderate	107 (41.8)	1755 (39.5)	1847 (42.9)	2604 (45.2)	1724 (46.2)	89 (45.4)		8126 (43.5)
Major	17 (6.6)	305 (6.9)	366 (8.5)	553 (9.6)	456 (12.2)	28 (14.3)		1725 (9.2)
Death	0	15 (0.3)	12 (0.3)	27 (0.5)	47 (1.3)	10 (5.1)		111 (0.6)
Substance								
Only cannabis	47 (18.4)	248 (5.6)	105 (2.4)	124 (2.2)	110 (2.9)	19 (9.7)	<.001	653 (3.5)
Others	209 (81.6)	4200 (94.4)	4202 (97.6)	5634 (97.8)	3623 (97.1)	177 (90.3)		18 045 (96.5)
Caller location								
Health care facility	234 (91.4)	4029 (90.6)	3947 (91.6)	5380 (93.4)	3498 (93.7)	184 (93.9)	<.001	17 272 (92.4)
Residence (own, other)	4 (1.6)	176 (4.0)	125 (2.9)	113 (2.0)	38 (1.0)	4 (2.0)		460 (2.5)
Other	18 (7.0)	241 (5.4)	233 (5.4)	260 (4.5)	195 (5.2)	8 (4.1)		955 (5.1)
Unknown	0	2 (0)	2 (0)	5 (0.1)	2 (0.1)	0		11 (0.1)
Route of exposure								
Inhalation	4 (1.6)	54 (1.2)	30 (0.7)	31 (0.5)	16 (0.4)	4 (2.0)	<.001	139 (0.7)
Ingestion	121 (47.3)	1423 (32.0)	1261 (29.3)	1669 (29.0)	1115 (29.9)	101 (51.5)		5690 (30.4)
Other	40 (15.6)	701 (15.8)	534 (12.4)	705 (12.2)	400 (10.7)	20 (10.2)		2400 (12.8)
Unknown	91 (35.5)	2270 (51.0)	2482 (57.6)	3353 (58.2)	2202 (59.0)	71 (36.2)		10 469 (56.0)

Abbreviation: NA, not applicable.

^a^ Percentages may not sum to 100% due to rounding.

^b^ *P* values indicate results determined using χ2 tests.

^c^ Minor outcomes involved some signs or symptoms but they were minimally bothersome and generally resolved rapidly. Moderate outcomes involved more pronounced, more prolonged, or more systemic symptoms. Major outcomes involve signs or symptoms that were life-threatening or resulted in major residual disability or disfigurement.^6^

Twice as many cases involved children (aged 5-13 years) in 2021 vs 2019 (3.1% vs 1.3%; *P* < .001). In 2021, 57.0% of the cases involved females vs 49.8% in 2019 (*P* < .001). Other substances were involved in 95.4% of 2019 cases vs 92.2% of 2021 cases (*P* < .001).

## Discussion

Intentional, suspected suicidal cannabis exposures reported to US poison centers increased from 2009 to 2021. Increases during and after the pandemic were notable and greatest among children and females. Most involved other substances; due to the cross-sectional nature of the data, we could not identify a causal association between cannabis use and a suicide attempt. Associations between cannabis use and mental health, especially among younger users, have been reported.^2^ Our findings suggest that deleterious medical outcomes occur frequently among older adults. Explanations could include interactions between age-related conditions and medications.

This study has limitations. Data from the NPDS rely on self-reported exposures by health care facilities or individuals, so reported exposures represent a subset of all exposures. Although case documentation is not verified beyond what is reported, NPDS coding maximizes quality and completeness of information. In addition, we were not able to examine individual, environmental, or contextual factors associated with suicidal ideation.

With more US states legalizing adult-use cannabis, increases in cannabis use will likely persist. It is important to further examine the suspected association between cannabis use and suicidal behaviors and how risks can be prevented or mitigated.

## Notes

Supplement 1. **eTable.** Generic Cannabis Codes for Case Identification in the National Poison Data System (NPDS), 2009-2021

Supplement 2.
Data Sharing Statement
