# Supplementary material for: Suspected Suicidal Cannabis Exposures Reported to US Poison Centers, 2009-2021
Source: JAMA Netw Open. 2023 Apr 19;6(4):e239044. doi: 10.1001/jamanetworkopen.2023.9044 (PMC10116359; doi:10.1001/jamanetworkopen.2023.9044)
Supplement: Supplement 1. — eTable. Generic Cannabis Codes for Case Identification in the National Poison Data System (NPDS), 2009-2021 [file jamanetwopen-e239044-s001.pdf]

Supplementary Online Content

Graves JM, Dilley JA, Klein T, Liebelt E. Suspected suicidal cannabis exposures reported to US poison centers, 2009-2021. *JAMA Netw Open*. 2023;6(4):e239044. doi:10.1001/jamanetworkopen.2023.9044

eTable. Generic Cannabis Codes for Case Identification in the National Poison Data System (NPDS), 2009-2021

This supplementary material has been provided by the authors to give readers additional information about their work.

© 2023 Graves JM et al. *JAMA Network Open.*

eTable. Generic cannabis codes for case identification in the National Poison Data System (NPDS), 2009-2021.

Generic Code	Description
0083000	Marijuana: Dried Plant
0310126	Marijuana: Other or Unknown Preparation
0310121	Marijuana: Edible Preparation
0310146	Cannabidiol (CBD)
0310124	Marijuana: Concentrated Extract (Including Oils and Tinctures)
0200618	Marijuana: Pharmaceutical Preparation
0310123	Marijuana: Undried Plant
0310096	eCigarettes: Marijuana Device Flavor Unknown
0310122	Marijuana: Oral Capsule or Pill Preparation
0310125	Marijuana: Topical Preparation
0310097	eCigarettes: Marijuana Liquid Flavor Unknown
0310033	eCigarettes: Marijuana Device Without Added Flavors
0310034	eCigarettes: Marijuana Device With Added Flavors

© 2023 Graves JM et al. *JAMA Network Open.*
