# Supplementary material for: Suspected Suicidal Cannabis Exposures Reported to US Poison Centers, 2009-2021
Source: JAMA Netw Open. 2023 Apr 19;6(4):e239044. doi: 10.1001/jamanetworkopen.2023.9044 (PMC10116359; doi:10.1001/jamanetworkopen.2023.9044)
Supplement: Supplement 2. — Data Sharing Statement [file jamanetwopen-e239044-s002.pdf]

Data Sharing Statement

Graves. Suspected Suicidal Cannabis Exposures Reported to US Poison Centers, 2009-2021. *JAMA Netw Open*. Published April 19, 2023. doi:10.1001/jamanetworkopen.2023.9044

Data
Data available: No

Additional Information
Explanation for why data not available: The data used in this study were obtained from the National Poison Data System (NPDS), the data repository for the American Association of Poison Control Centers. Researchers interested in obtaining these data can do so by request from AAPCC.
